# Use of Thymol in *Nosema ceranae* Control and Health Improvement of Infected Honey Bees

**DOI:** 10.3390/insects13070574

**Published:** 2022-06-24

**Authors:** Uros Glavinic, Jovan Blagojevic, Marko Ristanic, Jevrosima Stevanovic, Nada Lakic, Milorad Mirilovic, Zoran Stanimirovic

**Affiliations:** 1 Department of Biology, Faculty of Veterinary Medicine—University of Belgrade, Bul. Oslobodjenja 18, 11000 Belgrade, Serbia; jovan.blagojevic95@gmail.com (J.B.); mristanic@vet.bg.ac.rs (M.R.); rocky@vet.bg.ac.rs (J.S.); zoran@vet.bg.ac.rs (Z.S.); 2 Department of Statistics, Faculty of Agriculture—University of Belgrade, Nemanjina 6, 11080 Belgrade, Serbia; nlakic@agrif.bg.ac.rs; 3 Department of Economics and Statistics, Faculty of Veterinary Medicine—University of Belgrade, Bul. Oslobodjenja 18, 11000 Belgrade, Serbia; mija@vet.bg.ac.rs

**Keywords:** honey bee, *Nosema ceranae*, thymol, oxidative stress, immune-related gene expression

## Abstract

**Simple Summary:**

In the European Union, there is no registered product for the control of the honey bee endoparasite *Nosema ceranae*. Thus, researchers are looking for options for *Nosema* treatment. The aim of this study was to investigate the effect of a natural essential-oil ingredient (thymol) derived from *Thymus vulgaris* on honey bees infected with *N. ceranae.* Thymol exerted certain positive effects (increasing bee survival, immunity, and antioxidative protection), as well as positively affecting the spore loads in *Nosema*-infected bees. However, when applied to *Nosema*-free bees, thymol caused certain health disorders; therefore, beekeepers should be careful with its use.

**Abstract:**

*Nosema ceranae* is the most widespread microsporidian species which infects the honey bees of *Apis mellifera* by causing the weakening of their colonies and a decline in their productive and reproductive capacities. The only registered product for its control is the antibiotic fumagillin; however, in the European Union, there is no formulation registered for use in beekeeping. Thymol (3-hydroxy-p-cymene) is a natural essential-oil ingredient derived from *Thymus vulgaris*, which has been used in *Varroa* control for decades. The aim of this study was to investigate the effect of thymol supplementation on the expression of immune-related genes and the parameters of oxidative stress and bee survival, as well as spore loads in bees infected with the microsporidian parasite *N. ceranae*. The results reveal mostly positive effects of thymol on health (increasing levels of immune-related genes and values of oxidative stress parameters, and decreasing *Nosema* spore loads) when applied to *Nosema*-infected bees. Moreover, supplementation with thymol did not induce negative effects in *Nosema-*infected bees. However, our results indicate that in *Nosema*-free bees, thymol itself could cause certain disorders (affecting bee survival, decreasing oxidative capacity, and downregulation of some immune-related gene expressions), showing that one should be careful with preventive, uncontrolled, and excessive use of thymol. Thus, further research is needed to reveal the effect of this phytogenic supplement on the immunity of uninfected bees.

## 1. Introduction

Nosemosis is a honey bee disease caused by microsporidia from the genus *Nosema* (*N. apis* and *N. ceranae*). It is the most widespread microsporidian infection of adult *Apis mellifera* individuals that leads to chronic infection, the weakening of honey bee colonies [1], and the decline of their productive and reproductive capacities [2,3,4].

This endoparasite survives in the infected colony throughout the year and reaches its maximum number before the end of winter and in early spring [5,6]. Diseased bees excrete a large amount of infectious agent in their feces, which easily reaches uninfected bees. In intensive beekeeping, forager bees have high metabolic requirements, which further potentiates the development of nutritional and energy stress [1,7]. The only registered product for nosemosis is the antibiotic fumagillin; however, in the European Union, there is no formulation registered for use in beekeeping [8,9,10]. The tendency to reduce the use of antibiotics in beekeeping has led to the continual search for natural alternatives in the treatment of diseases such as nosemosis, reviewed in [11] including dietary supplements [12,13,14,15,16,17,18,19,20,21,22].

Similar to *Varroa* ectoparasite control [23,24], organic chemicals or natural-based treatments are welcomed for *Nosema* treatment [25,26,27]. Thymol (3-hydroxy-p-cymene) is a natural compound as it is a component of essential oil, derived from *Thymus vulgaris* L., Lamiaceae, and many other plant species. The inhibitory effect of thymol on the growth of pathogenic bacteria and fungi, such as *Escherichia coli*, *Streptococcus spp.*, *Salmonella typhimurium*, *Staphylococcus aureus*, *Aspergillus flavus*, and *Cryptococcus neoformans*, has been known for many years [28,29]. In beekeeping, thymol has been used for decades to control the honey bee mite *Varroa destructor* [30,31] with variable success [32]. The first studies of the potential effect of thymol in the control of *Nosema* infection in the hive were performed at the beginning of the 21st century [33,34], but to date, the anti-*Nosema* potential and effect of thymol on *Nosema* have not been fully elucidated. It was assumed that the mechanism of thymol action is based on penetration into the *Nosema* spore, interfering with the plasma membrane and preventing spore germination [28,33]. Thymol can be found naturally in low concentrations in honey [35,36], and it was thought not to leave residues in bee products [37]. However, recent studies revealed residues of thymol in honey [38] and beeswax [38,39].

Thymol is thought to interfere with the GABA signaling pathway in the central nervous system of insects [40], and there is therefore a legitimate concern that thymol also affects bees [41]. In addition, in a study by Bergougnoux et al. [42], thymol negatively affected the phototactic behavior of bees. Boncristiani et al. [43] reported that thymol had increased the susceptibility of bees to *N. ceranae* infection through the reduced expression of the *Dscam* and *Basket* genes, which are significant cellular and humoral immune factors, respectively, in defending bees from parasites [44,45]. In several studies, treatment with thymol (orally or topically) did not induce toxic effects on bees [42,46,47], and bees even lived longer compared with the control [47]. These data together with earlier observations on the low toxicity of thymol [48], as well as its importance for beekeeping, led to thymol’s approval by the European Union [49] for the control of the honey bee mite *V. destructor* in conventional and organic beekeeping [28,37].

An infection with *N. ceranae* induces oxidative stress in bees and compromises their immunity [13,14,15,16], particularly in combination with pesticides [50,51]. Thus, it could be important to identify the effects of a thymol-enriched diet on the biochemical and transcription levels of *Nosema*-infected bees. The aim of this study was to investigate the effect of thymol supplementation on bees’ spore loads, their expression of immune-related genes, and the parameters of oxidative stress, as well as the survival of bees infected with microsporidia *N. ceranae*.

## 2. Materials and Methods

### 2.1. Bees and Experimental Design

Colonies of *Apis mellifera* bees used as a bee brood source were located at the apiary of the Faculty of Veterinary Medicine, University of Belgrade. In accordance with good beekeeping practice, all colonies were without clinical symptoms of either adult bee diseases or brood diseases. The *Varroa* infestation was maintained at a minimum level, following the recommendations of the COLOSS BEEBOOK [52]. A sealed brood (prior to emergence) was taken from five randomly selected hives. The frames were placed in net bags to keep any emerging bees on the frame and left overnight in an incubator under controlled conditions (temperature 34 ± 1 °C, and humidity 66 ± 1%). After 12 h, the emerged bees were collected and placed randomly in cages that were specially designed for this purpose, following the method described by Glavinic et al. [13]. Each cage contained 80 randomly selected bees (15 for the RNA extraction, 15 for the analyses of oxidative stress, and 30 for counting *Nosema* spores, while the remaining 20 bees were used for survival monitoring). According to the experimental design (Table 1), the cages (experimental units) were divided into six experimental groups: bees in the non-infected control (NI), bees infected with *N. ceranae* (infected control—I), bees treated with thymol (treatment control—T), and three treatment groups—all infected with *Nosema* and treated with thymol from the first, third, and sixth day (I-T1, I-T3, and I-T6, respectively). The whole experiment was repeated, and the results were merged into a single dataset. 

The bees were fed ad libitum with 50% (*w*/*v*) sucrose solution. The tested substance was thymol (Sigma-Aldrich, St. Louis, MO, USA, CAS 89-83-8). The feeding solution was prepared in a concentration of 0.1 mg/g (0.1 g/kg) of syrup, according to Costa et al. [47]. The syrup volume was measured before and after the bees had been fed for 24 h to ascertain syrup consumption [16]. Further, the average consumption per bee per day was calculated. The dead bees were removed from cages daily, and the number of dead bees per cage was recorded. 

### 2.2. Experimental Infection with N. ceranae Spores

On the third day of the experiment, the bees from the infected control group (I) and the treatment groups (I-T1, I-T3, and I-T6) were infected according to the experimental design (Table 1), with inoculum freshly prepared according to a previously published procedure [15]. The final concentration of inoculum was 1 × 10^6^ spores/mL, while the presence of *N. ceranae* and absence of *N. apis* was confirmed by species-specific PCR tests [53]. The food was removed from the cages two hours before the infection was performed, in order to starve the bees and ensure better consumption of the inoculum.

### 2.3. Counting of Nosema Spores

The number of spores per bee was estimated according to the methodology adopted by Glavinic et al. [13]. Briefly, the abdomen of a single bee was placed in a 1.5 mL tube with 1 mL of dH_2_O and homogenate in TissueLyser II (Qiagen, Germany) for 1 min at 25 Hz. The suspension was observed using a hemocytometer according to the OIE guidelines [54].

### 2.4. Gene Expression Analyses

RNA was extracted from five bees from each cage, using a Quick-RNA MiniPrep Kit (Zymo Research, Irvine, CA, USA) according to the manufacturer’s instructions. For cDNA synthesis, 1000 ng of RNA per sample were reverse-transcribed using the RevertAid™ First Strand cDNA Synthesis Kit (Thermo Fisher Scientific, Vilnius, Lithuania).

The expression levels of abaecin, hymenoptaecin, defensin, apidaecin, and vitellogenin (immune-related genes) were determined by the methodology described in our previous studies [15,16]. The 2^-ddCt^ method was used, while β-actin was an internal control gene [55]. The median value of the non-infected group served as a calibrator.

### 2.5. Oxidative Stress Analyses 

The spectrophotometric analyses described in our previous study [15] were used for oxidative stress-parameter measurements: activities of the antioxidative enzymes superoxide dismutase (SOD), catalase (CAT), and glutathione S-transferase (GST), and the concentrations of malondialdehyde (MDA). Pools of five bees collected from every cage on each sampling day (6, 9, and 15) were used and analyzed on a UV/VIS Spectrophotometer BK-36 S390 (Biobase Jinan, Shandong, China,).

### 2.6. Statistical Analyses

The Kaplan–Meier survival function was used for the survival dynamic presentation. To compare the difference in survival between two or more independent groups, a log-rank test was used. To ensure heterogeneity of the data for gene expression levels and spore loads, we used the Mann–Whitney U test in order to determine the significance of the difference between medians of two samples. We used ANOVA to maintain homogeneity of the oxidative stress data when determining the significance in the differences between three or more means. Moreover, the Tukey’s test was used to test the difference between the means of sample pairs.

All conclusions were made by comparing the level of significance of the realized value of the sample test statistics, *p*, with standard levels of significance, 0.05 and 0.01.

The statistical analyses of the results were done with Statistica Software (StatSoft Inc., Tulsa, OK, USA).

## 3. Results and Discussion

**Figure 1:**
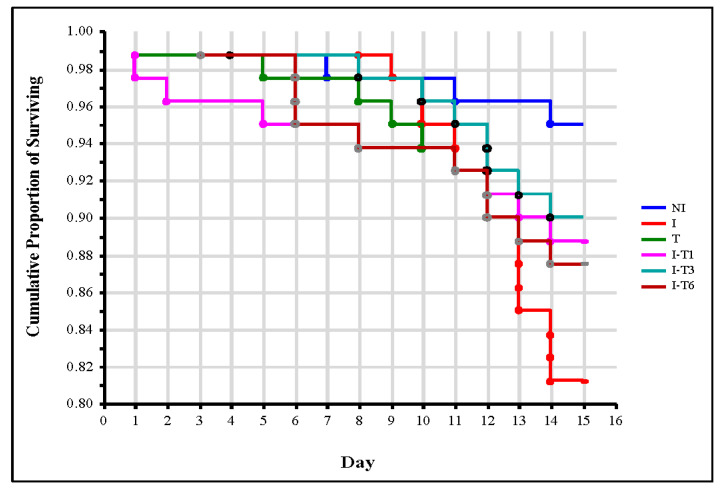
Survival curves of bees from control groups and from groups treated with thymol. Group infected with *N. ceranae* (I), group that was non-infected but treated with thymol (T), groups infected with *N. ceranae* and treated with thymol from day 1 (I-T1), day 3 (I-T3), and day 6 (I-T6), and non-infected (NI) group.

*Nosema ceranae* infection induced significant bee mortality (*p* = 0.008) in the infected control (I) group compared with the non-infected control (NI) group, confirming a negative impact of *Nosema* infection on bees’ lifespan [13,14,15,16]. When simultaneously analyzing the number of dead bees in the control group I and in all the thymol-treated groups, the log-rank test found an absence of significant differences (χ^2^ = 5.173; *p* = 0.270). Furthermore, the log-rank test for the two groups revealed the absence of statistically significant differences in the number of dead bees between control group I and each group treated with thymol (*p* ≥ 0.126) (Figure 1). The same *Nosema*- and thymol-induced mortality was reported by Maistrello et al. [28]. Bee mortality induced with thymol could be explained by the toxic potential of thyme essential oils being classified as a moderately toxic product [28]. On the other hand, in this experiment (Figure 1), there was no significant differences (log-rank test: χ^2^ = 3.048; *p* = 0.550) observed in the survival dynamic of the control group NI and groups treated with thymol. The number of dead bees between the control group NI and each group treated with thymol was not statistically significantly different (*p* ≥ 0.095). This result is in accordance with those of Ebert et al. [46], Costa et al. [47], and Bergougnoux et al. [42], who found that thymol was not toxic to bees. Nevertheless, according to EU Regulation 834/2007 on organic production [49], thymol is authorized for use in *Varroa* control in organic beekeeping. Keeping in mind that the presence of *V. destructor* reduces the potential of bees to combat *N. ceranae* [56], a great advantage of thymol could be its potential to control both pathogens.

**Figure 2:**
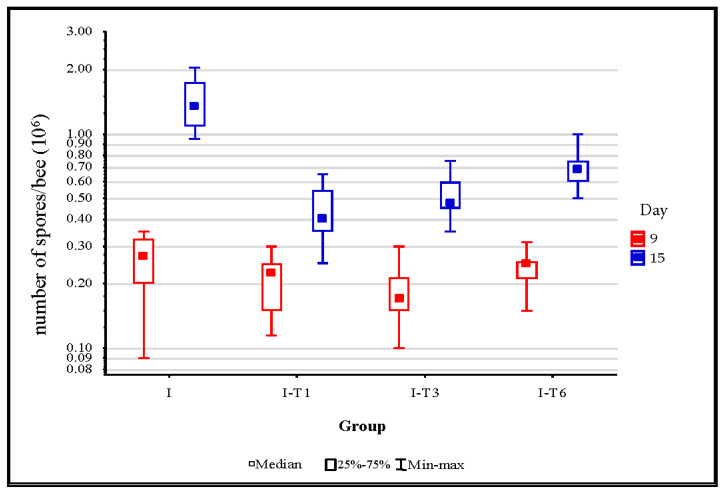
*N. ceranae* spore loads on day 9 and day 15 in bees from the control group and from groups infected and treated with thymol. Group infected with *N. ceranae* (I) and groups infected with *N. ceranae* and treated with thymol from day 1 (I-T1), day 3 (I-T3), and day 6 (I-T6).

According to the Mann–Whitney U test, no significant differences (*p* > 0.05) between the control group I and groups infected and treated with thymol (I-T1, I-T3, and I-T6) were noticed in the number of *Nosema* spores in bees collected on day 9 (semi-logarithmic diagram, Figure 2). On day 15, the number of spores were significantly higher (*p* < 0.01), and the numbers varied between the experimental groups from 0.2 to 2 × 10^6^/bee (semi-logarithmic diagram, Figure 2), which is similar to some previous studies [15,16,47,57,58]. A significantly higher (*p* < 0.001) *Nosema* spore load was detected in the infected control group compared with bees collected on day 15 from the I-T1, I-T3, and I-T6 groups. This difference could be due to thymol’s inhibitory effect on *Nosema* development, which resulted in lower *Nosema* spore loads in the latter stages of the experiment. Maistrello et al. [28] assumed that the mechanism of thymol’s anti-*Nosema* effect is based on its interaction with the *Nosema* spore by interfering with the plasma membrane and preventing spore germination [28,33]. Similar to our results, Costa et al. [47] detected significantly lower *Nosema* spore loads in bees supplemented with thymol, while Van den Heever et al. [59] reported that thymol decreased spore load by 40%. Keeping in mind the described effects of thymol consumption, especially its potential in *Nosema* control, we further investigated its impact on immune-related genes and the oxidative stress in bees infected with *Nosema*.

**Figure 3:**
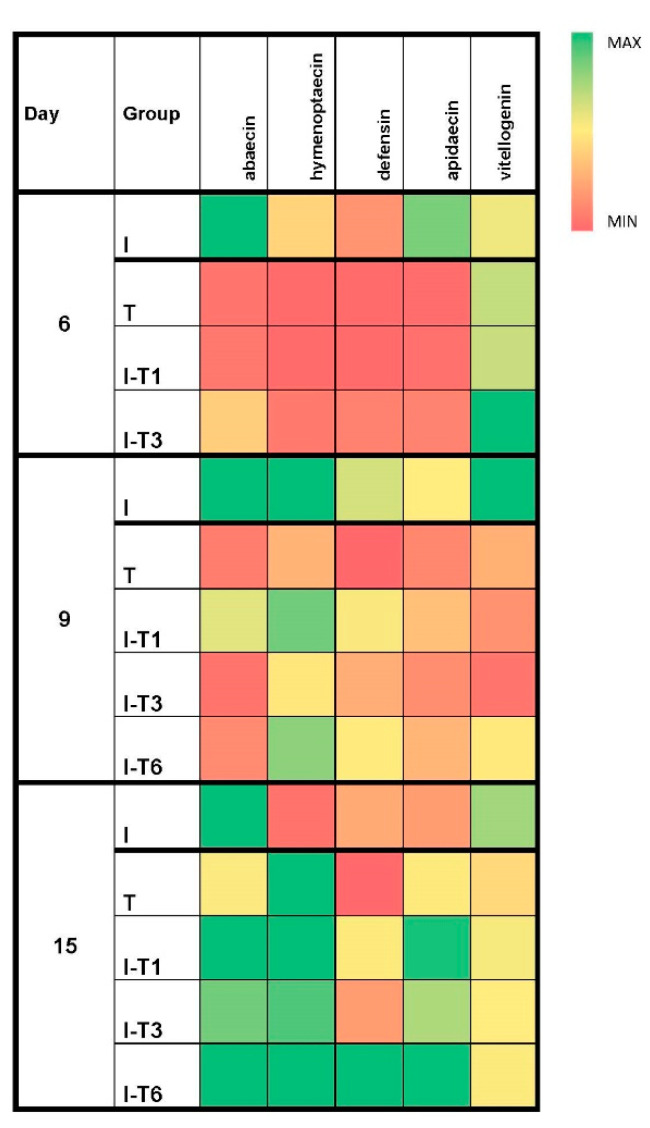
Heat map of median values for the relative genes’ expression levels (abaecin, hymenoptaecin, defensin, apidaecin, and vitellogenin) at different time points (day 6, 9, and 15) in the experimental groups. Group infected with *N. ceranae* (I), group that was non-infected but treated with thymol (T), groups infected with *N. ceranae* and treated with thymol from day 1 (I-T1), day 3 (I-T3), and day 6 (I-T6).

**Figure 4:**
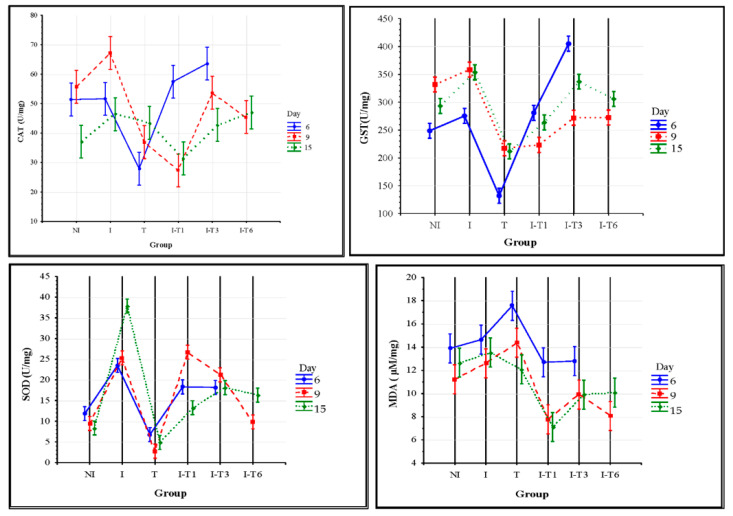
Activities of superoxide dismutase (SOD), catalase (CAT), and glutathione S-transferase (GST) and malondialdehyde (MDA) concentration at different time points in experimental groups. Non-infected control (NI) group, *N. ceranae*-infected control (I) group, thymol-treatment control group (T), and groups infected and supplemented with thymol from day 1 (I-T1), day 3 (I-T3), and day 6 (I-T6).

The expression levels of the abaecin gene on day 9 were significantly higher in the infected (I) group (Mann–Whitney U test: *p* ≤ 0.012) compared with all groups treated with thymol (Figure 3 and Figure S1). Therefore, thymol decreased abaecin’s gene expression more than the *Nosema* infection, which is in accordance with findings of Maistrello et al. [28] and Costa et al. [47], who identified that thymol worked best after longer usage (at the end of the experiment), contrary to *Nosema* whose negative effects increased over time [13,47]. Moreover, the oxidative stress parameters were inconsistent and without a clear pattern (Figure 4) in bees collected on day 9, which is in line with the detected number of spores on day 9 (that did not differ significantly among groups), because bees were struggling to overcome the negative impact of the endoparasite (*N. ceranae*) through the production of ROS (Reactive Oxygen Species) and by activating antioxidant (protective) mechanisms in order to prevent tissue damage caused by the ROS [14].

At the end of the experiment (on day 15), the thymol consumed through the sucrose syrup exerted the best anti-*Nosema* effect (observed through the number of spores, Figure 2). Moreover, the results of the activity and concentrations of monitored oxidative stress parameters revealed the best effect of thymol consumption on day 15 (Figure 4). The activities of all antioxidative enzymes (SOD, CAT, and GST) and MDA concentrations were significantly higher according to Tukey’s test (*p* < 0.05) in the infected (I) group compared with the majority of the other groups (Figure 4). The lowest activities of antioxidative enzymes (SOD, GST, and CAT) were detected in the groups supplemented with thymol from the first day of experiment (T and I-T1), which were lower than the majority of the other groups (Tukey’s test: *p* < 0.05). The reason for these findings could be the balance in the redox potential of thymol-fed bees and in the bees’ success in controlling the antioxidative response. In the infected control (I) group (which did not receive thymol), *N. ceranae* successfully induced oxidative stress, which was detected by increased levels of SOD, CAT, GST, and MDA at the end of the experiment (Figure 4). Oxidative stress induced by different stressors, such as *N. ceranae* [15,60] and environmental pollutants [61,62], was also reported in some previous studies.

The expression levels of the apidaecin and hymenoptaecin genes on day 15 were significantly higher (Mann–Whitney U test: *p* ≤ 0.021) in all thymol-treated groups compared with the infected control (I) group. Apidaecin-gene expression levels were lower (*p* ≤ 0.036) in the T group (thymol-treated and *Nosema*-free) compared with the infected and thymol-treated bees, while for hymenoptaecin expression levels, no difference was defected (*p* > 0.05). Bees from the T group had lower values for abaecin and defensin gene expression compared with groups infected and treated with thymol (I-T1, I-T3, and I-T6). The defensin levels in group T were lower (*p* ≤ 0.022) even when compared with the infected control group I (Figure 3 and Figure S1).

The number of *Nosema* spores at the end of the experiment (Figure 2) indicates an evident anti-*Nosema* effect of thymol in our cage experiment. Accordingly, the results of the gene expression levels showed that thymol prevented the suppressive effect of *N. ceranae* on the expression of immune-related genes. Moreover, thymol treatment reduced the activity of SOD, CAT, and GST, as well as concentration of MDA (Figure 4). The suppression of certain genes in the thymol-treated and *Nosema*-free group (T) indicates the potential immunosuppressive effect of thymol when given preventively to uninfected bees. The negative impact of thymol on some other insect species has been known; thus, thymol was used for the suppression of the development and survival of adult mosquitoes [63,64] and cockroaches [65]. Gene expression levels for all genes except vitellogenin continuously increased over the experiment throughout the three sampling times in all groups infected with *Nosema* and treated with thymol (I-T1, I-T3, and I-T6). These findings could be explained by the anti-*Nosema* activity of thymol [28,47], which subsequently mitigated the immunosuppression caused by *Nosema*.

The vitellogenin levels in bees collected on day 15 (Figure 3 and Figure S1) were the least altered. There was no difference in vitellogenin expression levels between the infected group (I) and the thymol-treated group (T) with to the other experimental groups (*p* > 0.05). This indicates that the level of vitellogenin was similar in the group that received thymol and in the groups that did not. Therefore, *N. ceranae* inhibited the expression of the vitellogenin gene equally to thymol. In addition, no synergistic effect (*Nosema* or thymol) on the vitellogenin gene expression was noted, keeping in mind that the same level of vitellogenin gene expression was obtained in the groups treated with thymol and infected with *Nosema* (I-T1, I-T3, and I-T6). Thymol used in *Varroa* treatment in the study of Boncristiani et al. [43] led to the downregulation of the vitellogenin gene, as well as other genes important for the detoxification and immunity of bees, which were not monitored in our experiment. Changes in the vitellogenin gene expression levels under the influence of thymol has been linked to possible modifications of bee-specific traits that are significantly influenced by vitellogenin [66]. It is also worth noting the rapid effect of thymol application on gene expression in the brain of the honey bee: significant upregulation of the transient-receptor-potential-like (TRPL) gene and downregulation of the octopamine receptor OA1 gene *Amoa1* [67].

Initial studies of the effect of thymol on bees infected with *Varroa* mites [31,46,68] did not report negative effects of thymol on bees. However, further research reported some negative effects of thymol on bees [43,69,70,71], which was one of the reasons for our research.

Our study revealed the positive effects of thymol on the health of *Nosema*-infected bees without producing negative effects. The proven anti-*Nosema* effect of thymol and subsequent prevention of *Nosema’s* negative effects could be beneficial on bees’ health. Moreover, our results indicate that in *Nosema*-free bees, thymol itself could cause certain disorders (side effects such as inducing oxidative stress, immunosuppression of the monitored genes, and reduction in bee longevity). Keeping in mind the obtained results, one should be careful with the preventive, uncontrolled, and excessive use of thymol. Further research should be conducted in order to determine the possible mechanisms of thymol activity when applied to infected and uninfected bees.

## Figures and Tables

**Table 1:** Experimental design.

Group ^1^	Initial Treatment Day ^2^	*N. ceranae* Infection Day ^2^	Sampling Day ^2^		
NI	-	-	6	9	15
I	-	3	6	9	15
T	1	-	6	9	15
I-T1	1	3	6	9	15
I-T3	3	3	6	9	15
I-T6	6	3	-	9	15

^1^ Bees were either non-infected (NI) or infected with *N. ceranae* (I) and treated with thymol (T). ^2^ Days after bee emergence.

## Data Availability

The data presented in this study are available upon request from the corresponding author. The data are not publicly available due to the excessive data size.

## Supplementary Materials

The following supporting information can be downloaded at: https://www.mdpi.com/article/10.3390/insects13070574/s1, Figure S1: Expression levels of abaecin, hymenoptaecin, defensin, apidaecin and vitellogenin at different time points (day 6, 9 and 15) in experimental groups. N. ceranae infected control (I) and groups infected and supplemented with thymol from day 1 (I-T1), day 3 (I-T3) and day 6 (I-T6).
