# Supplementary material for: Use of Thymol in Nosema ceranae Control and Health Improvement of Infected Honey Bees
Source: Insects. 2022 Jun 24;13(7):574. doi: 10.3390/insects13070574 (PMC9319372; doi:10.3390/insects13070574)
Supplement: Supplementary file 1 [file insects-13-00574-s001.zip › insects-1741273-supplementary.pdf]

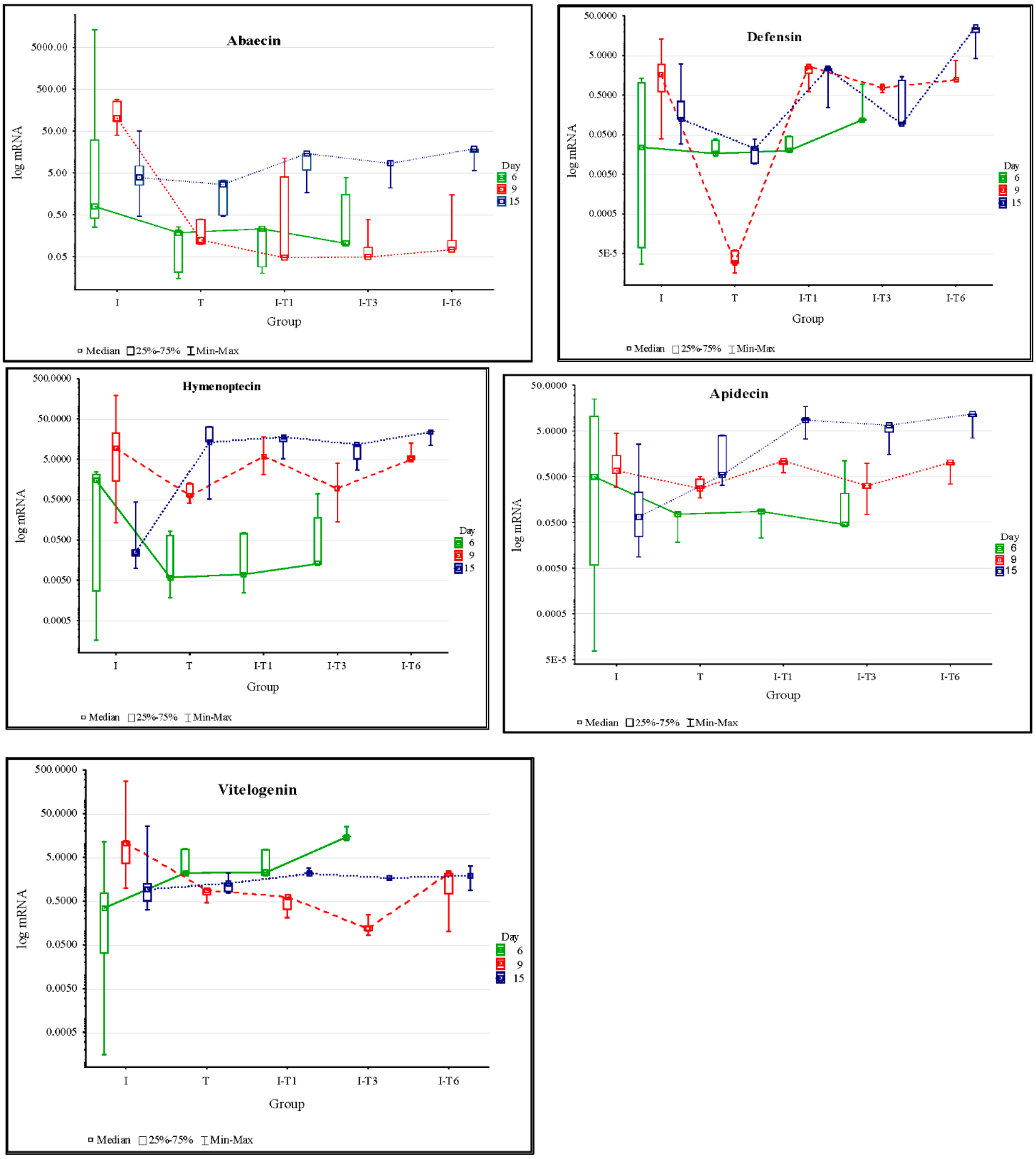

Figure S1. Expression levels of abaecin, hymenoptaecin, defensin, apidaecin and vitellogenin at different time points (day 6, 9 and 15) in experimental groups. *N. ceranae* infected control (I) and groups infected and supplemented with thymol from day 1 (I-T1), day 3 (I-T3) and day 6 (I-T6)
